# Implementation of health technology: Directions for research and practice

**DOI:** 10.3389/fdgth.2022.1030194

**Published:** 2022-11-10

**Authors:** JEWC (Lisette) van Gemert-Pijnen

**Affiliations:** Center for eHealth & Wellbeing Research, University of Twente, Enschede, Netherlands

**Keywords:** success, failures, implementation, health, technology, healthcare, digtal care

## Introduction

The success of health technologies in practice is highly dependent on the implementation approach. However, a lot of health technologies fail due to a lack of commitment and investment to introduce, maintain, and manage these technologies in a sustainable way (1). Implementation is often described as a complex process, involving a variety of factors that play a decisive role during the development process (2). To monitor and understand these factors, a holistic and multidisciplinary approach of implementation is demanded (3). Holistic because technologies are not just devices. Technologies create an infrastructure for the way of working, for new services and concepts on how to change and improve healthcare, how to align work practices with technologies, how to prepare healthcare workers to use technologies, how to engage stakeholders to invest in maintenance and how to assess the impact on healthcare? Multidisciplinary, only with knowledge and insights from different disciplines as social and behavioural sciences, engineering and business, user friendly, accessible, and affordable technologies can be realised. Therefore, implementation must deal with resources (e.g., time, staff, budget, investment policies), ethical concerns (privacy, security, regulations, ownership), governance (policy, accountability, responsibility etc.), and eSkills (capabilities, culture, etc.) (1, 2).

To better guide, monitor and understand the implementation, this paper describes and introduces directions for research and practice. A novel approach for implementation will be highlighted, and finally opportunities will be outlined for increasing knowledge on implementation.

## Views on implementation

Implementation can be described as a process of several planned and guided activities to launch, introduce and maintain technologies in a certain context to innovate or improve healthcare. These activities deliver the evidence for adoption and upscaling a technology in healthcare practices.

Based on knowledge, insights from research and lessons learned from practice (2) several key principles for implementation can be announced:

### Implementation is intertwined with development

Too often, implementation is seen as post-design activity that is planned and executed after the design of a technology is finished. However, current visions on technology development state that implementation plays an important role right from the start (2, 4, 5).

The development or redesign of a technology can be considered as the creation of an infrastructure for healthcare delivery to change or improve healthcare since it intervenes with existing care practices such as the division of labor, time, finance, legislation and regulations for using technology in a treatment or self-care process. Implementation starts with activities to get insights in the risks and factors that can influence the uptake and adoption. Therefore, systematic attention should be given to the implication for individuals, health care and society at large. Potential implementation issues such as limited resources (e.g., time, staff, and money), ethical (privacy, security, dependency, cultural diversity) or personal drawbacks (e.g., skills, motivation, and uncertainties) should be identified. These issues should also be accounted for in the subsequent cycles of development (prototyping, design and employment) (6). In this way, the well-known pitfalls of stakeholder disregard can be avoided.

### Engaging stakeholders is crucial

Stakeholders should be identified and involved at start and during the development of a technology to ensure engagement and to achieve commitment about resources, capacities, and maintenance. Key stakeholders can be identified based on their role in the process of implementation and the level of strategic influence they have on short- or long-term success and failure of implementation (7–9). Stakeholders help to create the technology by means of being involved in activities like identifying the values to be realized with a technology and discussing critical issues for implementation.

Involving end users and stakeholders from different backgrounds, with different interests and strategic influence (political, medical, policy, commercial) is important for creating trust, commitment, ownership and for organizing the resources, finance, and capacities for the technology. Different stakeholders often have different motives, goals and values for a technology. Having a thorough understanding of and appreciation for these motivations is important, however these discussions with stakeholders are often lacking, or not well-communicated (2). It is also important to prioritize the values of different stakeholders and make choices based on this prioritization. Value proposition maps and business models are needed to implement the infrastructures for care delivery through technology (10). Such a business model should address the societal, medical, commercial, ethical and socio-cultural aspects of using technologies in healthcare (2, 11, 12).

### Implementation needs continuous monitoring

To implement technologies in healthcare, continuous monitoring is needed to track and understand changes and impact on the healthcare system. Formative iterative data collections are relevant about real-time usage, adoption, and impact on care pathways, staff and costs. The iterative data collections provide evidence for implementation, *via* process methods combined with performance methods to establish cost-effectiveness, clinical outcomes and efficiency. For instance, methods to determine the effect of transferring hospital care to home care on health outcomes, or methods to estimate the differences technologies make in health care as compared to care as usual (13). Monitoring research should reach beyond the golden standard of randomized clinical trials. Robust methods are necessary to assess the full spectrum of potential benefits and risks a technology can have. For example, methods to track data about real time use of a technology, like logfiles, user feedback systems, a risk assessment to understand the pros and contras for the organization of care and a business model to define the potentials for maintenance and upscaling care (14).

### Governance is essential for sustainable implementation

Implementation is often an unofficial appointed task, no one is responsible for it. Lack of cooperation with managers of care organizations results in *ad hoc* planning and “reinventing the wheel”. Inappropriate planning, lack of commitment of staff, lack of resources induces an increase in workload and a negative attitude of staff towards digitalization of healthcare. Governance, referring to leadership, vision, policy and accountability is essential to create ownership and to invest in training and long-term data collections to understand the impact of technology on healthcare and society. Therefore, governance could facilitate participatory development discussing different perspectives of staff and management to create commitment, consensus and ownership and responsibilities for maintenance. Maintenance of technology is often underestimated, which can lead to obsolete technologies and in the end de-implementation.

Nowadays, new challenges are the validation and certifications of new and all existing health technologies under the new Medical Device Regulations (MDR) of which many aspects are still unknown (15). Careful planning is needed to fulfil the regulatory demands for healthcare technologies, to guarantee safety, security before the technologies go to the EU-market. It will be important for managers of healthcare organizations to understand the novel classification systems for changing or updating the medical devices, for planning, design, execution of impact assessments and for supporting the development of a quality management system. Knowing how other healthcare settings that have implemented similar technology can be very helpful and efficient (16). This will provide insights in interoperability and standardization, in accordance with international interoperability standards, data sharing, and local regulations (17).

The key principles reflect the complexity of implementation and the need for a holistic approach towards sustainable health technology implementation.

## Approaching a framework for implementation of health technology

A numerous models and frameworks have evolved that aim to understand the processes and driving factors involved in implementation, and to predict outcomes (2, 4, 5). The frameworks and models have different perspectives. For example, frameworks like, RE-AIM Framework (18), Consolidated Framework for Implementation Research (19) were introduced to implement clinical and medical based interventions using evidence from research findings. These frameworks express the acceptance and adoption of research findings in practice. In this view, implementation refers to a set of planned, intentional activities that aim to put into practice evidence-based practices in real-world services, with the goal to benefit end-users of these services. However, these frameworks do not focus on the capacities and characteristics of technology to change, innovate healthcare and how technology could be integrated into workflow and care pathways (2, 6).

Considering the aforementioned views on implementation, a process driven development guideline is in preparation, building on the CEHRES roadmap and business modelling (2, 6, 11, 12). The implementation guideline can be considered as a maturity scan (20) to guide and to assess the process and outcomes of implementation. The maturity scan entails 5 domains: users, stakeholders, organization, system (What are the expectations, and requirements of the end users, stakeholders, management of the organization and the technical system surrounding the proposed innovation?); legal, ethical, privacy aspects and regulations (What are the legal, ethical and technical considerations regarding implementation of a technology); business model (what are the expected cost/benefits, resources, capacities to implement technology); economic aspects (is a technology affordable, sustainable considering stakeholders and market); effectiveness (is a technology equally or more effective compared to current practice). Each domain refers to several questions and methods to assess the maturity of a certain domain. The end scores visualizes (a spider plot*)* the status quo of implementation and specifies what has to been done to improve the implementation process (directions, and methods). This scan will be further validated to develop a holistic and multidisciplinary based implementation approach (20).

## Increasing knowledge on health technology implementation

The section focusses on implementation of health and medical technologies. To maximise the impact of technology on healthcare and society advanced methods are needed to acquire quantitative and qualitative data to validate technologies according to law- and regulations for medical devices. I advocate a crossing border approach, as on the edge of different disciplines new concepts for implementation and methodologies will emerge. Therefore, I welcome interdisciplinary and multidisciplinary research, to stimulate the use of novel and comprehensive health technology implementation assessments and advanced methods to engage stakeholders, creating added value for patients, healthcare, and society.
